# Chronic psychosocial stressors are associated with alterations in salience processing and corticostriatal connectivity

**DOI:** 10.1016/j.schres.2018.12.011

**Published:** 2019-11

**Authors:** Robert A. McCutcheon, Michael A.P. Bloomfield, Tarik Dahoun, Mitul Mehta, Oliver D. Howes

**Affiliations:** a Department of Psychosis Studies, Institute of Psychiatry, Psychology & Neuroscience, Kings College London, De Crespigny Park, London SE5 8AF, UK; b Psychiatric Imaging Group, MRC London Institute of Medical Sciences, Hammersmith Hospital, London W12 0NN, UK; c Institute of Clinical Sciences, Faculty of Medicine, Imperial College London, London, W12 0NN, UK; d Translational Psychiatry Research Group, Research Department of Mental Health Neuroscience, Division of Psychiatry, University College London, 6th Floor, Maple House, 149 Tottenham Court Road, London WC1T 7NF, UK; e Clinical Psychopharmacology Unit, Research Department of Clinical, Educational and Health Psychology, University College London, 1–19 Torrington Place, London WC1E 6BT, UK; f National Institute of Health Research University College London Hospitals Biomedical Research Centre, University College Hospital, Euston Road, London W1T 7DN, UK; g The Traumatic Stress Clinic, St Pancras Hospital, 4 St Pancras Way, London NW1 0PE, UK; h Department of Psychiatry, University of Oxford, Warneford Hospital, Oxford, OX37 JX, UK; i Department of Neuroimaging, Institute of Psychiatry, Psychology & Neuroscience, Kings College London, De Crespigny Park, London SE5 8AF, UK

**Keywords:** Stress, Schizophrenia, Psychosis, Striatum, Corticostriatal, Functional connectivity

## Abstract

Psychosocial stressors including childhood adversity, migration, and living in an urban environment, have been associated with several psychiatric disorders, including psychotic disorders. The neural and psychological mechanisms mediating this relationship remain unclear. In parallel, alterations in corticostriatal connectivity and abnormalities in the processing of salience, are seen in psychotic disorders. Aberrant functioning of these mechanisms secondary to chronic stress exposure, could help explain how common environmental exposures are associated with a diverse range of symptoms. In the current study, we recruited two groups of adults, one with a high degree of exposure to chronic psychosocial stressors (the exposed group, n = 20), and one with minimal exposure (the unexposed group, n = 22). All participants underwent a resting state MRI scan, completed the Aberrant Salience Inventory, and performed a behavioural task – the Salience Attribution Test (SAT). The exposed group showed reduced explicit adaptive salience scores (cohen's d = 0.69, p = 0.03) and increased aberrant salience inventory scores (d = 0.65, p = 0.04). The exposed group also showed increased corticostriatal connectivity between the ventral striatum and brain regions previously implicated in salience processing. Corticostriatal connectivity in these regions negatively correlated with SAT explicit adaptive salience (*r* = −0.48, p = 0.001), and positively correlated with aberrant salience inventory scores (*r* = 0.42, p = 0.006). Furthermore, in a mediation analysis there was tentative evidence that differences in striato-cortical connectivity mediated the group differences in salience scores.

## 1 Introduction

Several environmental factors that can be considered chronic psychosocial stressors are associated with an increased risk of developing schizophrenia, but the psychological and neurobiological mechanisms mediating this increased risk remain incompletely understood (Tost et al., 2015). Disruption in salience processing has been proposed as a central deficit in schizophrenia, whereby the ‘salience’ of a stimulus refers to the significance that stimulus holds for an organism (Winton-Brown et al., 2014). Corticostriatal circuits play an important role in salience processing, and disruption of these circuits is seen in schizophrenia (Dandash et al., 2014; Fornito et al., 2013; Levitt et al., 2017). In the current study, we investigated whether exposure to chronic psychosocial stressors was associated with alterations in salience processing, and whether this was linked to changes in corticostriatal connectivity.

### 1.1 Chronic psychosocial stressors and schizophrenia

Many of the environmental risk factors associated with schizophrenia can be broadly conceptualised as chronic psychosocial stressors (Selten and Cantor-Graae, 2005; Walker and Diforio, 1997). These include childhood adversity, migration, and urbanicity (De Loore et al., 2007; Howes et al., 2017; Linscott and van Os, 2013; Morgan et al., 2009; Van Nierop et al., 2014).

Studies investigating the influence of these psychosocial stressors upon brain function have typically investigated one factor at a time, despite the fact that the risk factors cluster together and potentially share common underlying mechanisms (Hjern et al., 2004; Morgan et al., 2007; Wicks et al., 2005). Moreover, epidemiological evidence suggests that there are at least additive, and potentially synergistic effects between risk factors (Guloksuz et al., 2015; Lataster et al., 2012; Morgan et al., 2014; Schafer and Fisher, 2011).

**Fig. 1:**
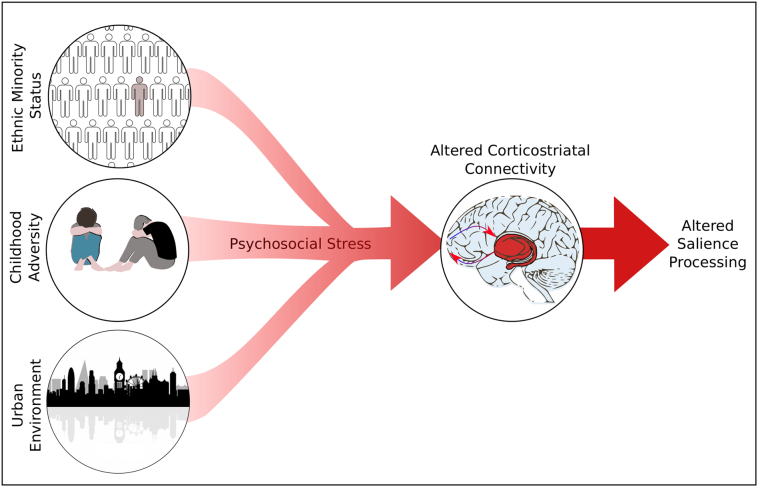
Proposed pathway in which exposure to chronic psychosocial stressors leads to alterations in corticostriatal connectivity and subsequent alterations in salience processing.

Several lines of evidence have suggested links between psychosocial stress exposure and alterations in salience processing. The finding that individuals exposed to early life trauma show evidence of both blunted responses to reward, and increased rates of psychotic experiences, suggests that alterations to both adaptive and aberrant salience processing mechanisms may be present (Croft et al., 2018; Hanson et al., 2016). Given the central role of dopamine in salience processing, the finding that presynaptic dopamine function appears altered in immigrants and individuals that have suffered childhood adversity, suggests that this may be a mechanism via which these exposures lead to an increased risk of mental illness (Egerton et al., 2017, Egerton et al., 2016; Howes et al., 2017; Pruessner et al., 2004). There is also evidence that exposure to chronic psychosocial stressors is associated with functional alterations in brain regions involved in salience processing (Akdeniz et al., 2014; Lederbogen et al., 2011; McCutcheon et al., 2018b; Teicher et al., 2016), and to corticostriatal functional connectivity (Hanson et al., 2018; Hart et al., 2018). (Hanson et al., 2018; Hart et al., 2018). Taken together this suggests that psychosocial stress exposure could lead to cortico-striatal dysfunction and aberrant salience processing (Fig. 1). To our knowledge, however, none have directly investigated how these functional alterations relate to salience processing.

### 1.2 Salience processing

The Salience Attribution Test (SAT) was developed to quantify four related aspects of salience processing. The test involves participants responding to a probe that is paired with stimuli that vary across two dimensions (e.g. colour and shape). One of these dimensions is associated with a greater probability of reward (the outcome relevant dimension), whereas the other bears no relationship to the likelihood of reward (the outcome irrelevant dimension). The test has both implicit (based on reaction times) and explicit (based on participant ratings) components, and measures both aberrant (the extent to which the irrelevant dimension is thought to signal outcome) and adaptive salience (the extent to which relevant dimension is thought to signal to outcome).

Studies using the SAT in individuals with schizophrenia, have consistently demonstrated reduced adaptive salience compared to healthy controls (Abboud et al., 2016; Katthagen et al., 2016; Pankow et al., 2016; Roiser et al., 2009). Differences in aberrant salience have also been observed (Katthagen et al., 2016; Pankow et al., 2016), but this is not a consistent finding (Abboud et al., 2016; Roiser et al., 2009). Abnormal salience processing has also been demonstrated in those at high clinical risk of psychosis, and in dependent cannabis users (Bloomfield et al., 2016; Roiser et al., 2013); while in healthy controls greater dopamine synthesis capacity within the ventral striatum has been linked to higher aberrant salience scores (Boehme et al., 2015). The aberrant salience inventory (ASI) is a questionnaire that was developed in order to quantify the subclinical phenomenology of disrupted salience processing (Cicero et al., 2010). Inventory scores are related to measures of psychosis proneness (Cicero et al., 2010), and have been found to be increased in individuals treated with dopamine agonists (Poletti et al., 2014).

Several neural systems are involved in salience processing. Schultz et al. demonstrated that mesostriatal dopamine neurons play a role in assigning value to stimuli, based on accompanying rewards (Schultz et al., 1997), while more recent research has shown that these neurons respond to surprising stimuli even in the absence of any change in value, suggesting that their role extends beyond value encoding to include signalling the salience of relevant stimuli in general (Takahashi et al., 2017; Winton-Brown et al., 2014). In concert with the role that striatal dopamine plays, cortical regions are also involved in the propagation of salience signals. Appropriate functioning of corticostriatal connections is necessary for the successful integration of these cortical networks and striatal dopamine signalling (Menon and Uddin, 2010; Peters et al., 2016; Roiser et al., 2010), and disruption of corticostriatal connectivity has been observed in disorders of salience processing such as schizophrenia (Dandash et al., 2014; Fornito et al., 2013; Levitt et al., 2017).

In the current study, we recruited individuals with a history of either high or low exposure to chronic psychosocial stressors. Corticostriatal connectivity was measured using resting state functional magnetic resonance imaging (rfMRI), and participants also completed the Aberrant Salience Inventory and undertook the SAT. We hypothesised that exposed individuals would display increased aberrant, and reduced adaptive salience scores. We also hypothesised that exposed individuals would display alterations in corticostriatal functional connectivity, and that these alterations would be related to alterations in salience processing.

## 2 Materials and methods

### 2.1 Participants

Two groups of healthy volunteers were recruited, one that had been exposed to chronic psychosocial stressors (the ‘exposed’ group), and one with minimal exposure (the ‘unexposed’ group). Participants were recruited in person, and via online, leaflet and newspaper advertising. Two groups of healthy volunteers were recruited, one that had been exposed to chronic psychosocial stressors (the ‘exposed’ group), and one with minimal exposure (the ‘unexposed’ group). Participants were recruited via online, leaflet and newspaper advertising. Online and newspaper advertising was performed in both and rural areas, while leaflet advertising was performed only in rural areas. Group assignment was made only following the screening interview. All subjects were aged 18–45, had no personal history of psychiatric illness, and no family history of psychotic illness. All participants provided written informed consent, and the study had research ethics committee permission.

The exposed group had exposure to at least two of the following three risk factors: Childhood and current dwelling in urban environment (defined as local authority population density > 50 persons/ha); a history of 1st or 2nd generation migration; and a history of childhood adversity before age 16 years, which was defined as a Childhood Trauma Questionnaire subscale (physical, emotional, or sexual abuse) classification score of “moderate/severe” or “severe/extreme” (Bernstein et al., 2003). The unexposed group currently lived in a non-urban area, had no history of urban living for longer than six months, had no history of migration, and no history of childhood adversity.

Population density of current dwelling was obtained from the 2011 census (Office of National Statistics, 2011). Questionnaires conducted included the Aberrant Salience Inventory (ASI) (Cicero et al., 2010), and the Childhood Trauma Questionnaire (Bernstein et al., 2003).

### 2.2 Salience attribution test

Aberrant and adaptive salience was measured using the SAT (Roiser et al., 2009). This is a task in which participants are presented with an image from 1 of 4 possible categories (blue animal, red animal, blue household object, red household object), this image is immediately followed by a probe which the participant must respond to as rapidly as possible to maximise potential monetary reward (the magnitude of which is proportional to speed of response). One dimension of the pre-probe category is relevant to the probability of reward (e.g. if colour is the relevant dimension, 90% of probes following a red image would be accompanied by a reward, in contrast to 10% of probes following a blue image), while the other is irrelevant (e.g. in the previous case, both animals and household objects would have a 50% chance of being followed by a reward).

Two runs (64 trials each) were performed. Results are obtained for adaptive (relevant) and aberrant (irrelevant) salience, both based on participant reported estimated probabilities on visual analogue scales (SAT explicit salience) and reaction times (SAT implicit salience). SAT explicit adaptive salience represents the extent to which participants report a reward as more likely to follow following the relevant stimuli, compared to irrelevant stimuli. SAT implicit adaptive salience is a measure of how much more quickly a participant reacts to stimuli associated with reward in the relevant dimension. Measures of SAT aberrant salience relate to how much more likely participants rate or respond across the irrelevant dimension. For detailed methods see previously published reports (Roiser et al., 2009, Roiser et al., 2013).

SAT implicit aberrant, SAT explicit aberrant, and SAT implicit adaptive scores, and aberrant salience inventory scores were skewed and therefore square root transformed prior to analysis as previously recommended (Roiser et al., 2009).

### 2.3 Demographic and behavioural data analysis

All statistical analysis was performed using R version 3.3.2. The normality of variables was checked using the Shapiro-Wilks test, and results were square root transformed if skewed (Roiser et al., 2009). The significance of differences between groups for continuous variables was determined using an independent samples *t*-test. Pearson's χ2 test was used to test for group differences regarding categorical variables.

### 2.4 Resting state functional magnetic resonance imaging

#### 2.4.1 rfMRI: Data acquisition

Imaging data was acquired using a Philips 3 T Intera magnetic resonance imaging system. A ten-minute resting state scan was performed using a T2∗ weighted transverse echo planar image sequence (TR = 3000 ms, TE = 30 ms, flip angle = 90°, slice thickness = 3.25 mm, 2.19 mm × 2.19 mm in plane resolution, 44 slices, 200 volumes). A T1 structural image was then obtained with a gradient-echo scan (TR = 9.7 ms, TE = 4.6 ms, flip angle = 90°, slice thickness = 1.20 mm, 0.94 × 0.94 mm in plane resolution, 150 slices).

#### 2.4.2 rfMRI: Seed definition

Striatal seeds consisting of bilateral 3.5 mm radius spheres were placed in the inferior ventral striatum, superior ventral striatum, dorsal caudate, dorsal rostral putamen, dorsal caudal putamen, and ventral rostral putamen. These predefined seeds were initially reported by Di Martino et al. (2008) and have been repeatedly used in investigations of striatal connectivity (DelDonno et al., 2017; Fornito et al., 2013; Gabbay et al., 2013; Sarpal et al., 2016).

#### 2.4.3 rfMRI: Preprocessing

fMRI data was analysed using the CONN toolbox (version 17) implemented in SPM12 (Whitfield-Gabrieli and Nieto-Castanon, 2012). A standard preprocessing pipeline was used consisting of slice timing correction, realignment, and normalisation to MNI space based on segmentation parameters derived from segmentation of the T1 structural image. Images were smoothed with a Gaussian kernel of 6 mm full-width-half-maximum.

The Artifact Detection Tools (ART) toolbox (www.nitrc.org/projects/artifact_detect) was used to account for motion and artifact detection using anatomical component based correction (aCompCor) of temporal confounds relating to head movement and physiological noise. This method models noise effects at a voxel level based on estimates derived from principal components of noise regions of interest (white matter and CSF, eroded by one voxel to minimise partial volume effects), and then removes these from the BOLD timeseries using linear regression, global signal regression is not performed. Six residual head motion parameters and their first order temporal derivatives were also entered as regressors into the first level model, as was an effect accounting for magnetisation stabilisation and its first order derivative. Artifact/outlier scans (average intensity deviated >5 standard deviations from the mean intensity in the session, or composite head movement exceeding 0.9 mm from the previous image) were also regressed out. Preprocessed data were temporally bandpass filtered (0.008–0.09 Hz).

#### 2.4.4 rfMRI: Connectivity analysis

Voxel wise connectivity maps for each participant were then derived by computing Pearson correlations between the signal average over each seed region, and the signal at each voxel over the entire brain. These were then converted to normally distributed Fisher's Z maps to allow second level general linear model analyses. At the second level, connectivity maps between exposed and unexposed groups were contrasted with each other for each seed (left and right hemisphere seeds were entered into the same model, so six group level comparisons were performed in total). Clusters were considered statistically significant if they passed height thresholds of p < 0.001 and cluster-level thresholds of p < 0.05 FWE-corrected.

### 2.5 rfMRI relationship with salience measures

Fisher transformed correlation coefficients were extracted from significant clusters and averaged (weighted by cluster size) for each of the seeds showing statistically significant results. Pearson correlation coefficients were then calculated between these connectivity values and participant salience scores, with Spearman correlations also performed to ensure results were not outlier driven. We next performed an exploratory analysis investigating whether the difference between exposed and unexposed groups' salience scores might be mediated by altered corticostriatal connectivity. Where we had observed a significant bivariate relationship between corticostriatal connectivity and salience scores we tested a mediation model using the R package ‘mediation’ using quasi-Bayesian MonteCarlo simulation (10,000 simulations) to test for significance (Tingley et al., 2009).

## 3 Results

### 3.1 Participant demographics

22 unexposed and 20 exposed participants were recruited; demographics are displayed in Table 1. No significant differences existed between groups for age or sex. As expected, measures of childhood trauma, population density and migration and were significantly different between the exposed and unexposed group.

**Table 1:** Demographic details of participants, salience scores, and MRI movement.

	Unexposed (n = 22)	Exposed (n = 20)	p-Value
Age (years), mean (SD)	26.3 (±6.5)	27.2 (±7.1)	0.67^b^
Sex n (%female)	11 (50.0%)	11 (55.0%)	0.99^c^
Aberrant Salience Inventory^a^	1.8 (±1.6)	2.7 (±1.3)	0.044^b^
Childhood Trauma Questionnaire	29.2 (±5.4)	36.0 (±9.0)	0.005^b^
Population Density (persons per hectare)	21.2 (±17.5)	81.2 (±32.5)	< 0.001^b^
% 1st Gen. Migrant	0 (0.0%)	7 (35.0%)	0.009^c^
% 2nd Gen. Migrant (both parents)	0 (0.0%)	9 (45.0%)	0.002^c^
% 2nd Gen. Migrant (one parent)	3 (13.6%)	3 (15.0%)	1.00^c^
Mean Motion	0.2 (±0.1)	0.1 (±0.1)	0.76^b^
Maximum Motion	1.4 (±2.3)	1.7 (±2.3)	0.69^b^
Valid Volumes	193.1 (±14.2)	195.6 (±7.3)	0.49^b^

a Square root transformed.

b Independent samples *t*-test.

c Chi-square test.

### 3.2 Salience measures

**Fig. 2:**
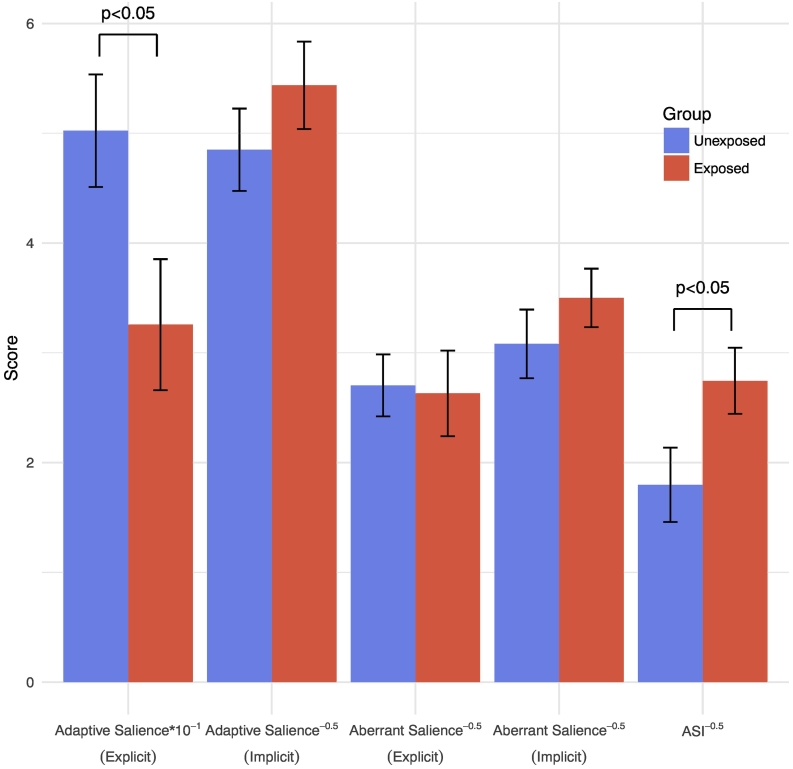
Salience attribution test, and aberrant salience inventory scores in exposed and unexposed individuals. Error bars (±1SE) ASI – Aberrant Salience Inventory.

The exposed group displayed significantly reduced SAT explicit adaptive salience (Cohen's d = 0.69, p = 0.03, df = 40), and increased aberrant salience inventory scores (d = 0.65, p = 0.04, df = 40) compared to the unexposed group (see Fig. 2). There were no other significant differences between groups on SAT measures. Within the whole group, SAT explicit aberrant and SAT explicit adaptive salience were negatively correlated (r_p_ = −0.44, p = 0.004, df = 40). There were no other statistically significant correlations between items of the SAT, nor between the SAT items and the ASI.

### 3.3 rfMRI data

**Fig. 3:**
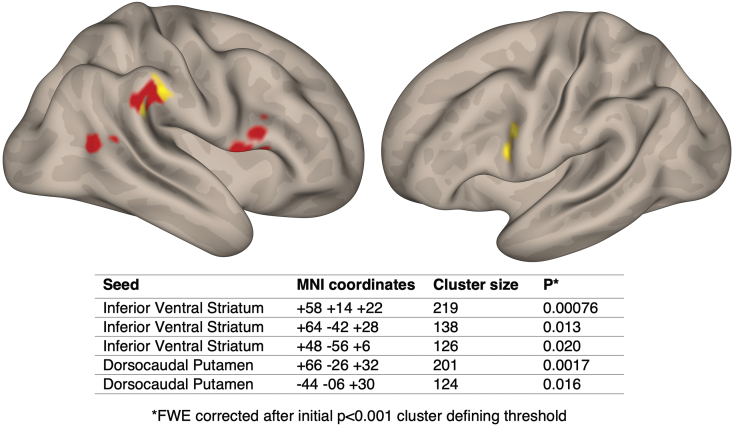
Areas of increased corticostriatal connectivity in the exposed compared to unexposed group. Red clusters relate to the seed in the inferior ventral striatum, and yellow represents the dorsocaudal putamen seed. (For interpretation of the references to colour in this figure legend, the reader is referred to the web version of this article.)

There were no differences between groups in terms of motion during the scan (see Table 1). Compared to the unexposed group, the exposed group showed increased connectivity between the inferior ventral striatum and three clusters (see Fig. 3). These clusters were centred on the right supramarginal gyrus, insular operculum and middle temporal gyrus, and the largest would survive Bonferroni correction for the six seeds examined (0.05/6 = p < 0.0083). The dorsocaudal putamen also displayed increased connectivity in the exposed group, with significant clusters centred on the right supramarginal gyrus and left insular operculum. The largest of the dorsocaudal putamen clusters would also pass Bonferroni correction. No significant clusters were identified for the other seeds. No seeds displayed increased connectivity in the unexposed group.

### 3.4 rfMRI relationship with salience measures

To investigate the potential behavioural relevance of the imaging findings, we averaged the connectivity z values within the significant clusters for each of the seeds (weighted by cluster size) to give a value for both average inferior ventral striatum connectivity, and dorsocaudal putamen connectivity. We then investigated correlations between these two summary connectivity measures and the behavioural measures.

**Fig. 4:**
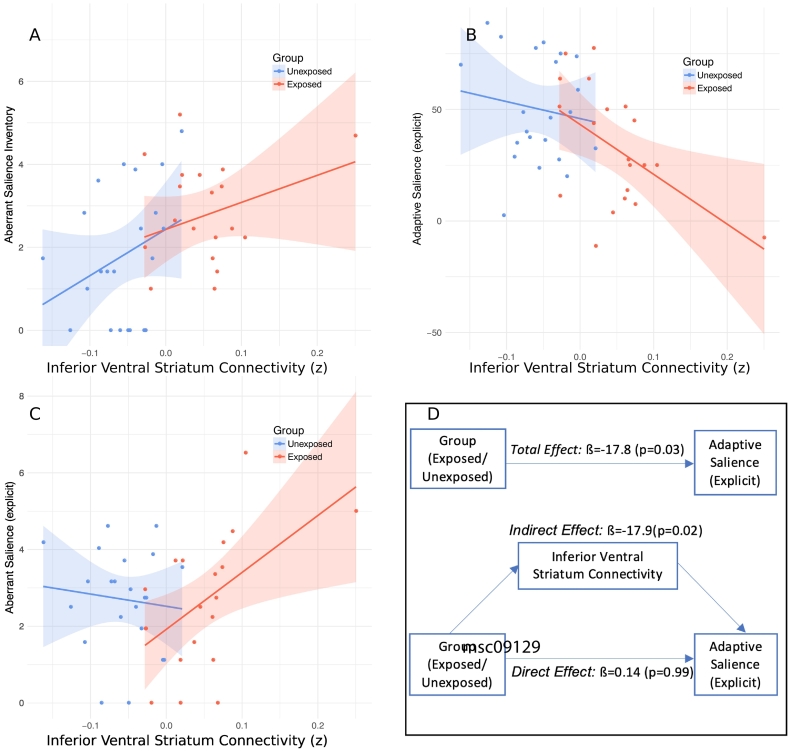
Relationship between salience scores and inferior ventral striatum connectivity. (A) Inferior ventral striatum connectivity correlates with explicit adaptive SAT scores in both the whole sample (r_p_ = −0.48, p = 0.001) and exposed individuals (rp = −0.52, p = 0.001). (B) Inferior ventral striatum connectivity correlates with explicit aberrant SAT scores in exposed individuals (r_p_ = 0.523, p = 0.016). (C) Inferior ventral striatum connectivity correlates with Aberrant Salience Inventory Scores in the whole sample (r_p_ = 0.42, p = 0.0056). (D) Mediation analysis – the relationship between risk factor exposure (i.e. whether participants in the exposed or unexposed group) and reduced SAT adaptive salience appears to be mediated by altered inferior ventral striatum connectivity.

Within the whole group inferior ventral striatum connectivity negatively correlated with SAT explicit adaptive salience scores (r_p_ = −0.48, p = 0.001, df = 40, see Fig. 4A) and this was also present within the exposed group alone (r_p_ = −0.52, p = 0.018, df = 18). Inferior ventral striatum connectivity and SAT explicit aberrant salience scores were negatively correlated within the exposed group (r_p_ = 0.523, p = 0.016, df = 18, see Fig. 4B), but not across the whole group. Inferior ventral striatum connectivity also positively correlated with Aberrant Salience Inventory scores across the whole group (r_p_ = 0.42, p = 0.0056, df = 40, see Fig. 4C). All these correlations remained significant when using Spearman's coefficient, indicating statistical significance was not outlier driven.

Dorsocaudal putamen connectivity also showed a negative correlation with SAT explicit adaptive salience scores (r_p_ = −0.36, p = 0.020, df = 40) across the whole group, and a positive correlation with SAT explicit aberrant salience only in the exposed group (r_p_ = 0.50, p = 0.025, df = 18). When using Spearman's coefficient the whole group correlation remained significant but the exposed group did not (r_s_ = 0.43, p = 0.059).

We next performed an exploratory mediation analysis to investigate whether the relationship between risk factor exposure (i.e. the binary variable of group) and salience scores might be mediated by altered corticostriatal connectivity. In the case of the inferior ventral striatum this showed a significant mediation effect for explicit adaptive SAT scores (indirect path estimate ß = −17.8, 95% CI = −34.5, −3.2, p = 0.02; direct path estimate ß 0.14, CI = −20.01, 20.87, p = 0.99, see Fig. 4D), and aberrant salience inventory scores (indirect path estimate ß = 4.5, 95% CI = 0.35, 9.26, p = 0.03; direct path estimate ß −0.885, CI = −6.65, 5.02, p = 0.77). The indirect path estimate between dorsocaudal putamen connectivity and explicit adaptive SAT scores was not significant (ß = −9.6, 95% CI = −27.9, 7.4, p = 0.28).

## 4 Discussion

In the current study, we demonstrated reduced adaptive salience in individuals that had been exposed to chronic psychosocial stressors, and found that this was related to increased connectivity between striatal seeds and cortical regions involved in salience processing. We also found increased scores on the aberrant salience inventory in the exposed group, but contrary to our initial hypotheses did not detect any between group differences on the aberrant or implicit measures of the SAT.

The exposed group demonstrated reduced SAT explicit adaptive salience, which represents a relative impairment in the learning of stimulus-reward associations. Dopamine neurons projecting to the ventral striatum play a central role in this process (Schlagenhauf et al., 2013; Schultz et al., 1997), and impaired dopaminergic reward signalling secondary to chronic stress has been put forward as one of the neurochemical alterations contributing to affective (Cabib and Puglisi-Allegra, 2012; Chen et al., 2015) and psychotic disorders (Howes et al., 2017). Reduced adaptive salience has been previously demonstrated in psychosis, and it was initially suggested that this primarily existed as a result of treatment with dopamine antagonists (Roiser et al., 2009). However, studies in un-medicated individuals at high risk of the disorder have displayed a similar pattern (Roiser et al., 2013; Schmidt et al., 2016), and some models suggest that although psychosis is associated with increased aberrant dopamine signalling (McCutcheon et al., 2018a), this may be accompanied by reductions in adaptive signalling (Maia and Frank, 2017). The exposed group also demonstrated increased scores on the ASI but not on any of the SAT measures of aberrant salience. This is similar to several studies in clinical populations, where it appears that the measure of SAT explicit adaptive salience is the most sensitive to group differences (Abboud et al., 2016; Katthagen et al., 2016; Pankow et al., 2016; Roiser et al., 2009). In terms of the magnitude of effect, the effect size observed in the current study for the measure of explicit adaptive salience (d = 0.69), was smaller than what has been observed in schizophrenia (d = 1.08 (Roiser et al., 2009)), but larger than what was reported in a recent study of individuals at clinical high risk of schizophrenia (d = 0.25, (Roiser et al., 2013)). It may be that alterations in adaptive salience processing occur more readily in the face of chronic stress, while increases in aberrant salience scores are only observed in established illness. The lack of group differences in implicit measures is something that has also been observed in some patient cohorts, and the correct interpretation of these measures remains unclear (Abboud et al., 2016; Neumann and Linscott, 2018; Roiser et al., 2013).

The exposed group also displayed increased functional connectivity between the ventral striatum and several cortical regions. A number of these clusters overlap with cortical areas that make up the *cingulo*-*opercular* or *salience* network (Gordon et al., 2016). This network is involved in the detection of relevant stimuli and the coordination of switching to appropriate brain states (Uddin, 2015), and shows an association with striatal dopamine function (McCutcheon et al., 2018c). Increased connectivity of the ventral striatum has also been observed in individuals with schizophrenia (Fornito et al., 2013), those at increased risk of the disorder (Dandash et al., 2014; Fornito et al., 2013), and those specifically affected by hallucinations (Rolland et al., 2015). In contrast to the current results, two of these studies simultaneously observed a pattern of reduced connectivity with the dorsal striatum (Dandash et al., 2014; Fornito et al., 2013). The pattern of connectivity alterations observed in the exposed group, therefore overlapped to an extent with what has been previously observed in schizophrenia, but also showed significant differences.

The environmental exposures, neural circuits, and cognitive mechanisms we studied have all been implicated in schizophrenia, but have transdiagnostic relevance, and as such may be best interpreted as mechanisms of general relevance to psychopathology rather than being disorder specific (Insel et al., 2010). Increased functional connectivity of the ventral striatum in has also been linked to an increased risk of subsequently developing depression (Pan et al., 2017), although findings in those with the established disorder have been inconsistent (Furman et al., 2011; Gabbay et al., 2013). This may be secondary to pathophysiological heterogeneity, illustrated by the fact that increased ventral striatum connectivity has been specifically observed in a high anxiety subgroup (Drysdale et al., 2016), while reduced connectivity has been observed in a high inflammation subgroup (Felger et al., 2016).

Recent work has also found that both early life adversity (Hanson et al., 2018), and economic disadvantage (Marshall et al., 2018) are associated with increased functional connectivity of the ventral striatum; although earlier work demonstrated a reduction in ventral striatum connectivity in those raised in households of lower parental education (Gianaros et al., 2011). Another study reported reduced functional connectivity of the caudate and putamen during an error monitoring task but did not use a ventral seed (Hart et al., 2018). Studies of urbanicity and migration have not directly examined corticostriatal connectivity, although mesolimbic signalling has been shown to relate to urban living (Kramer et al., 2017), and altered activation of the ventral striatum has been demonstrated in migrant individuals (Akdeniz et al., 2014).

We found that differences in ventral striatum connectivity mediated the association between chronic social stress and both reduced adaptive salience scores, and increased aberrant salience inventory scores. While the whole striatum appears to play a role in salience processing (Ilango et al., 2014; Oyama et al., 2015), it is the ventral striatum that has been principally implicated in studies using the SAT (Boehme et al., 2015; Roiser et al., 2010), and it is the ventral striatum that shows alterations in dopamine function in studies of early life stress (Oswald et al., 2014; Pruessner et al., 2004). It is, however, the associative striatum that displays the most marked dopaminergic dysfunction in schizophrenia (McCutcheon et al., 2018a), and the precise mechanism through which corticostriatal connectivity contributes to salience attribution is yet to be fully elucidated, although it is likely that dopaminergic mechanisms contribute (Bell et al., 2015; Boehme et al., 2015; Nagy et al., 2012; Roiser et al., 2010).

The study of risk factor exposure in individuals free of psychiatric illness means that the effect of exposure is not obscured by the presence of disease; however, a limitation is that it also raises the possibility that observed differences are markers of resilience as opposed to sequelae of exposure.

Psychosocial stress is a multidimensional construct and as a result we studied multiple stressors. The threshold used for defining groups in the case of migration was based on the finding that both first and second generation immigrants have an increased risk of psychosis,(Bourque et al., 2011), childhood adversity thresholds were based on previous studies (Kraan et al., 2017; Philip et al., 2014), while our cutoff for urbanicity was based was on census data for population densities in London suburbs. It may have been beneficial to specify even lower cutoffs regarding population density for the unexposed group, although this may have impeded recruitment. Our approach aimed to maximise the distance between groups in terms of exposure, however the resulting collinearity of risk factor exposures means that we were unable to determine the extent to which each individual exposure drives the observed group differences. While a limitation, this is typically an issue in single exposure studies as well (albeit a less explicit one), given that these do not tend to measure other potential risk factors, and the fact that these stressors show a tendency to cluster (Hjern et al., 2004; Morgan et al., 2007; Wicks et al., 2005).

A further potential limitation is the fact that apart from the inverse correlation between the two explicit SAT measures no significant association was observed between the other salience measures. This has been previously studied, and while this may be a result of the various measures capturing different aspects of salience processing it also suggests caution may be required when interpreting the meaning of these measures (Neumann and Linscott, 2018). In addition, the results of the mediation analysis only just reached statistical significance, and should be viewed with caution given the relatively low sample size for this form of analysis.

Future research has the potential to address several of the study's limitations. Longitudinal follow up can clarify whether observed behavioural and neurobiological differences relate primarily to resilience or risk. Studies in patient populations, also have the potential to determine the pathophysiological relevance of our findings. Future studies might consider a factorial design, which would enable testing as to specific effects of individual exposures, and whether additive and synergistic effects exist. Even when considering only three risk factors, however, eight possible combinations exists and a large sample would therefore be required. Alternatively using a continuous exposure score would be a powerful approach, although while recent scores have been proposed these remain to be validated (Padmanabhan et al., 2017; Vassos et al., 2018). Studies using positron emission tomography may allow for the investigation of whether the connectivity and behavioural differences observed involve dopaminergic alterations.

In conclusion, we found evidence that exposure to chronic psychosocial stressors was associated with alterations in salience processing and corticostriatal connectivity. Longitudinal studies may help probe the implications that this holds for the development of psychiatric disorders. In addition, future studies using multimodal methodologies are necessary to further understand the relationship between dopaminergic systems and functional connectivity.

## Conflict of interest

O.H. has received investigator-initiated research funding from and/or participated in advisory/speaker meetings organised by Astra-Zeneca, Autifony, BMS, Eli Lilly, Heptares, Jansenn, Lundbeck, Lyden-Delta, Otsuka, Servier, Sunovion, Rand and Roche. Neither Dr. Howes or his family have been employed by or have holdings/a financial stake in any biomedical company. M.M. has consulted for Cambridge Cognition, Lundbeck and Forum Pharmaceuticals in the past 3 years. He has also received research funding from Takeda, Eli Lilly and Roche. The other authors declare no competing financial interests.

## Role of the funding source

R.A.M.'s work is funded by the Wellcome Trust (no. 200102/Z/15/Z). T.D. was supported by a EU-FP7 MC-ITN IN-SENS grant (no. 607616) and by the National Institute for Health Research (NIHR) at Oxford Health NHS Foundation Trust. M.A.P·B is supported by a UCL Excellence Fellowship and the National Institute for Health Research (NIHR) University College London Hospitals Biomedical Research Centre. This study was funded by Medical Research Council-UK (no. MC-A656-5QD30), and Wellcome Trust (no. 094849/Z/10/Z) grants to O.H. and the National Institute for Health Research (NIHR) Biomedical Research Centre at South London and Maudsley NHS Foundation Trust and King’s College London. The views expressed are those of the authors and not necessarily those of the NHS, the NIHR or the Department of Health.

## CRediT authorship contribution statement

**Robert A. McCutcheon:** Conceptualization. **Michael A.P. Bloomfield:** Investigation. **Tarik Dahoun:** Formal analysis. **Mitul Mehta: Oliver D. Howes:** Writing - review & editing.

## CRediT authorship contribution statement

**Robert A. McCutcheon:** Conceptualization. **Michael A.P. Bloomfield:** Investigation. **Tarik Dahoun:** Formal analysis. **Mitul Mehta: Oliver D. Howes:** Writing - review & editing.
